# Testing for COVID-19: willful ignorance or selfless behavior?

**DOI:** 10.1017/bpp.2020.15

**Published:** 2020-05-08

**Authors:** LINDA THUNSTRÖM, MADISON ASHWORTH, JASON F. SHOGREN, STEPHEN NEWBOLD, DAVID FINNOFF

**Affiliations:** 1 Department of Economics, University of Wyoming, Laramie, WY, USA; 2 Department of Economics, University of Wyoming, Laramie, WY, USA; 3 Department of Economics, University of Wyoming, Laramie, WY, USA; 4 Department of Economics, University of Wyoming, Laramie, WY, USA; 5 Department of Economics, University of Wyoming, Laramie, WY, USA

## Abstract

Widespread testing is key to controlling the spread of COVID-19. But should we worry about self-selection bias in the testing? The recent literature on willful ignorance says we should – people often avoid health information. In the context of COVID-19, such willful ignorance can bias testing data. Furthermore, willful ignorance often arises when selfish wants conflict with social benefits, which might be particularly likely for potential ‘super-spreaders’ – people with many social interactions – given people who test positive are urged to self-isolate for two weeks. We design a survey in which participants (*n* = 897) choose whether to take a costless COVID-19 test. We find that 70% would take a test. Surprisingly, the people most likely to widely spread COVID-19 – the extraverts, others who meet more people in their daily lives and younger people – are the *most* willing to take a test. People's ability to financially or emotionally sustain self-isolation does not matter to their decision. We conclude that people are selfless in their decision to test for COVID-19. Our results are encouraging – they imply that COVOD-19 testing may succeed in targeting those who generate the largest social benefits from self-isolation if infected, which strengthens the case for widespread testing.

## Introduction

COVID-19 rapidly developed into a pandemic, and by 26 March 2020, the USA had the highest reported number of infected people in the world. A general message from public health experts is that effective control of the spread of COVID-19 requires widespread medical testing (WHO, 2020). The testing will serve to determine whether people are infected or not, and ideally also if they have been infected and have reached immunity status.

Three reasons motivate widespread testing. First, if a person learns that they are infected, they can take appropriate measures to reduce the probability of infecting others, such as the recommended 14-day self-isolation (Harvard Medical School, 2020). Second, the data provided by widespread testing will better inform the need for the current social distancing policies (e.g., sheltering at home, avoiding gatherings of 10 or more people, keeping at least 6 feet away from other people and temporarily closing schools, universities, daycare centers, major sports leagues, cultural events and public spaces) (Stock, 2020). Third, testing provides data about the asymptomatic rate in the USA (the share of infected people who show no or very mild symptoms) and insight into how close Americans are to developing herd immunity to COVID-19. This information is useful in order to determine when and where it makes sense to relax these costly social distancing measures.

While the USA has increased its capacity to conduct more testing, around 1.8% of the population had been tested by 30 April 2020 (COVID Tracking Project, 2020). The effectiveness of testing in controlling COVID-19 depends largely on how the tests are conducted. The ideal scenario is to test everyone, but that is infeasible. A second-best scenario is random sample testing (Stock, 2020). But for random testing to be effective, all sampled people would either need to voluntarily agree to be tested (which is unlikely, as we explain) or be forced to do so (which is illegal in the USA). The third-best (and first-best *feasible*) strategy is voluntary random testing. This strategy, however, could lead to a systematic selection bias – we will only test those individuals who prefer to learn their health status regarding COVID-19; a significant fraction of people might not want to know. These individuals might find that their private costs outweigh any social benefits from not infecting others. This implies that they might want to avoid testing. If their private costs include above-average opportunity costs of social interactions, then individuals who decline to be tested may also be disproportionately likely to become *super-spreaders*.

To understand why this might happen, consider the ongoing literature on willful ignorance of health information (also called strategic ignorance). While standard economic theory suggests people never ignore information that enables them to adjust behavior (Stigler, 1961), many new studies find that people willfully ignore medical diagnoses, even when such knowledge would enable them to adjust behavior to better accommodate their health condition (Sharot & Sunstein, 2020). For example, we see willful ignorance in many people at risk for breast cancer (Thompson *et al.*, 2002), Alzheimer's disease (Cutler & Hodgson, 2003), HIV (Hightow *et al.*, 2003) and Huntington's disease (Oster *et al.*, 2013). The study by Ganguly and Tasoff (2017) is particularly relevant: they observe people will avoid a costless test for herpes – a disease for which there is currently no cure, but for which information is useful in that it helps adjust behavior. People have also been found to willfully ignore health risk information, such as calories in food (Thunström *et al.*, 2016; Woolley & Risen, 2018; Sunstein, 2019; Thunström, 2019; Nordström *et al.*, 2020). Willful ignorance of health outcomes is likely to arise when people are torn between what they think they should do and what they want to do (Thunström, 2016; Woolley & Risen, 2018), or when ignorance allows them to form optimal expectations (downplay the probability of a bad health outcome; Oster *et al.*, 2013; Nordström *et al.*, 2020). For instance, a person may think she should eat healthy, but want to indulge in ice-cream—she might then choose to avoid learning about the exact amount of calories in the ice-cream in order to avoid either her inner pressure to reduce the ice-cream consumption or the guilt from consuming it despite being aware of the calorie content.

In this paper, we explore self-selection in COVID-19 testing in the USA. We examine if people willfully avoid getting tested for COVID-19, and, if so, what individual or household characteristics and circumstances are associated with testing avoidance. Given that random voluntary testing is not yet available in the USA, there are no observational data to rely on for our analysis. We therefore design a hypothetical randomized controlled trial (RCT). We recruit a nationally representative sample of 1000 participants. The study entails two treatments, across which we vary information about the potential emotional cost of testing before asking if participants would agree or disagree to a financially costless COVID-19 test. In the baseline treatment, we inform participants that if they are found to be infected, they are urged to self-isolate at home for 14 days. In the high-cost treatment, we tell participants that those who test positive are strongly urged to self-isolate, which may be in a self-quarantine site away from home.

We assume the test itself is costless and that the only cost incurred from a COVID-19 test is the recommended self-isolation for 14 days should the test come back positive. If people are concerned only with their private benefits and costs from taking a COVID-19 test, those with large private benefits and low private costs from knowing that they are infected will be the most likely to get tested. Private benefits are significant for people at elevated risk for severe health consequences if they contract the virus or with family members at higher risk, while private costs are low for those who generally live a solitary life, professionally and in private.

We expect elderly and those who have – or have a family member that has – pre-existing conditions to be more willing to test. Furthermore, we expect those at the lowest risk of losing out financially (e.g., risk to labor income or health care costs) or emotionally from self-isolating (i.e., if they are introverts who attach a low value to social interactions) to be the most willing to get tested for COVID-19. In contrast, if people are concerned only with social benefits and costs, we would expect those most at risk of exposing others to be the most likely to get tested (e.g., potential ‘super-spreaders’; i.e., people with jobs that entail mixing with other people, people living in urban areas, young people and people who are extroverts and attach a high value to social interactions).

People with the potential to be super-spreaders might be torn about learning whether they are infected by COVID-19. Both private costs and social benefits from being urged to self-isolate for the next 14 days (Harvard Medical School, 2020) might be high for this group, and so influence their testing decision in opposite directions. For example, consider the behavior of an extrovert who values social interactions highly. If unsure of being infected, the extrovert behaves just as if she is not infected (e.g., as found for Huntington's disease, by Oster *et al.*, 2013). Self-isolation means this person needs to give up highly valued social interactions – a factor that might deter them from a voluntary test for COVID-19. At the same time, their self-isolation provides particularly meaningful private *and* social health benefits from reduction in exposure to, and spread of, the disease. These benefits to learning whether they are infected might encourage them to voluntarily test. It is an open question if the benefits outweigh the costs, causing the extrovert to take a costless COVID-19 test. Similarly, imagine a store clerk who risks losing income if self-isolating for 14 days. The potential private loss of income would deter them from testing, while the social benefits from reducing disease spread would encourage taking the test. Again, the decision to test becomes an open question. Previous studies show that willful ignorance arises when prosocial behavior is privately costly (Dana *et al.*, 2007; Conrads & Irlenbusch, 2013; Onwezen & van der Weele, 2016; Gigerenzer & Garcia-Retamero, 2017; Grossman & van der Weele, 2017).

In this study, we find that around 70% of people want to take a costless COVID-19 test. As might be expected, people who worry more about their health are particularly likely to want to take the test. We also find that those *most* likely to want to take the test are those most likely to spread the virus if unaware of their infection, including young people and extroverts with a preference for socializing. Extroverts might also be at the highest risk for being infected, but even when we control for personal risks (amount of social interactions and worry about own health), we find they are more willing to test. The ability to afford to self-isolate for 14 days does not seem to affect the willingness to test. Our results suggest that there is a significant amount of selflessness in the decision to test; people appear to be highly concerned about the social benefits from testing for COVID-19 and little concerned about private costs.

In addition, we do not find the expected treatment effect of our experimental manipulation of the private cost to testing (i.e., the location of self-isolation (at home or in a facility away from home) does not seem to matter to the testing decision). Our expectation was that willful ignorance would be higher if self-isolation might take place away from home, since we assume self-isolation away from home is perceived as more costly. One interpretation of the lack of expected treatment effect is that it lends further support to the idea that private costs play a negligible role in the decision to test for COVID-19.

Our results matter because they underscore the value of widespread COVID-19 testing, even if such testing cannot be done randomly. Our findings suggest that widely available and costless voluntary testing will target rather than scare off those most likely to be ‘super-spreaders’.

## Methods and data

To test people's willingness to take a financially costless COVID-19 test, we designed a hypothetical field experiment. The experiment is a RCT with a between-subjects design, consisting of two treatments. In the first treatment (*treatment baseline*), participants were told they would be urged to self-isolate at home, if having tested positive. In the second treatment (*treatment high cost*), they were told they might be urged to self-isolate at a special site away from home.

Participants (*n* = 1000) were recruited by the research firm Qualtrics, and the sample was required to be nationally representative along the dimensions of gender, age, education, race, income and residential region (east, west, north or south). While the recruitment costs from Qualtrics are higher than when recruiting from Amazon Mechanical Turk or Turk Prime, Qualtrics continuously quality checks participants, which enabled us to avoid many issues that may otherwise contaminate online panels (e.g., see Chandler & Paolacci, 2017; Sharpe Wessling *et al.*, 2017). Participants received standard Qualtrics compensation to participate in a survey.

The sequence of the experimental study was as follows:

*Step 1:* All participants were asked screening questions at the front end of the survey about their gender, age, education, race, income and region in order to ensure the sample met US national quotas for those characteristics.

*Step 2:* Participants were asked whether they had already been tested for the COVID-19. If ‘yes’, they were asked why they got tested, the outcome of the test, how many days prior to survey participation they had taken the test and if the test was costly. If ‘no’, they were randomized into one of the two treatments and asked about their willingness to take a test.

Specifically, if in *treatment baseline*, they received the following information:

Currently, US authorities are working to test more people for the coronavirus. Legislators are urging people who test positive, i.e., are found to be infected by the virus, to self-isolate at home for 14 days.

If in *treatment high cost*, they were instead told:

Currently, US authorities are working to test more people for the coronavirus. Legislators are urging people who test positive, i.e., are found to be infected by the virus, to self-isolate for 14 days. Some states have started building self-quarantine sites – sites where people who have the virus would be isolated for 14 days. If people stay at those sites, it is easier to ensure they comply with the guidelines to self-isolate.

Thereafter, participants in both treatments were asked:

If you were given the opportunity to take a coronavirus test for free within the next 3 days, would you take the test?

Default alternatives to get or not get information have been shown to affect observed choices of ignorance (Grossman, 2014). To avoid nudging participants toward any particular answer, there was no default alternative; participants needed to choose either “Yes, I would take the test,” or “No, I would not take the test.”

*Step 3:* All participants were asked about their current level of social distancing (how many people outside their household they had been within 6 feet of in the last 3 days; how many gatherings with more than 10 people they had participated in; and self-assessed level of compliance with social distancing). They were also asked whether they supported the public recommendations for social distancing in general.

*Step 4:* Participants were asked questions about factors that might affect the perceived cost of a positive COVID-19 test (implying social isolation for 14 days), as well as the perceived benefits from being able to make behavioral adjustments. They were asked about their job situation, job security and possibility of the main income provider in the household taking sick leave; risk factors for contracting the virus (e.g., living in a urban area, working in a health care facility, working in a grocery store or pharmacy); risk factors (for self or any children) for suffering severe health consequences if contracting the virus (e.g., underlying health conditions that increases the risk, such as cancer, obesity, diabetes, etc.); level of extraversion (Francis *et al.*, 1992); and social lifestyle.

*Step 5:* Participants were asked about religious belonging, religiosity, political affiliation and social and fiscal conservatism (Everett, 2013).

The full survey can be found in the Online Supplementary Material.

Of our total sample, 103 participants stated that they had already been tested, while 897 stated that they had not already been tested. We asked those who had been tested for the primary reason they had taken the COVID-19 test. Table 1 shows their answers. As expected, given the current prevailing strategy in the USA of focusing the limited testing on people who are symptomatic, most people got tested because they themselves showed symptoms (almost 55%) or because someone close to them either showed symptoms or was diagnosed with COVID-19 (around 35%). Summary statistics for the 103 participants who had been tested before participating in our study are shown in the Online Supplementary Material.

**Table 1.** The primary reason for having been tested for COVID-19.

Reason	Number of participants	Percentage of all who had been tested
I showed symptoms	55	53.4
A family member showed symptoms	14	13.6
A friend showed symptoms	11	10.7
I wanted to know if I was infected	6	5.8
A family member tested positive	1	1.0
A friend tested positive	11	10.7
Other	5	4.6
Total	103	100

Our analysis focuses on the 897 participants who stated that they had not been tested for COVID-19. Due to a coding error in the survey at the beginning of the data collection, seven participants did not respond to the question on whether they were a business owner, employed or unemployed. We dropped these seven participants from our analysis, and we were left with 890 observations. Table 2 presents the summary statistics for these participants. Unless otherwise stated, all of the remaining analysis focuses on the results for this group of participants.

**Table 2.** Descriptive statistics.

Variable	Observations	Mean	SD	Minimum	Maximum
Female	890	0.527	0.495	0	1
Age	890	46.65	16.11	12	89
High-risk age	890	0.161	0.367	0	1
Rural area	890	0.348	0.477	0	1
Emotional tolerance	890	0.865	0.342	0	1
Financial tolerance	890	0.842	0.365	0	1
Lifestyle impact – healthy	890	0.920	1.233	0	5
Lifestyle impact – unhealthy	890	2.264	1.661	0	6
Business owner	890	0.100	0.300	0	1
Employed	890	0.536	0.499	0	1
Unemployed	890	0.364	0.481	0	1
Business impact	89	0.064	0.245	0	1
Employer impact	477	0.307	0.461	0	1
Social distant compliant – 6 feet	890	5.763	12.48	0	75
Social distant compliant – groups	890	0.473	1.074	0	5
Self health risk	890	0.955	1.309	0	10
Child health risk	276	0.067	0.397	0	7
Worry own health	890	1.501	0.991	0	3
Insurance	890	0.691	0.462	0	1
Republican	890	0.339	0.474	0	1
Democrat	890	0.432	0.496	0	1
Other political party	890	0.229	0.421	0	1
Extrovert	890	0.452	0.498	0	1

Table 2 shows that 53% of participants who had not yet been tested for COVID-19 are *Female*. The variable *Age* describes a participant's age in years, and the mean age in our sample is 47 years. The variable *High-risk age* is a dummy variable that takes the value 1 if a person is aged 65 years or older. Table 2 shows that 16% of our participants are aged 65 years or older. The variable *Rural area* takes a value 1 if participants stated that they live in a rural area and 0 if they live in an urban area. Table 2 shows that 35% of our participants live in a rural area.

The variables *Emotional tolerance* and *Financial tolerance* are dummy variables that take the value 1 if the participant answered that the maximum time (from the time of taking the survey) he/she would be able to emotionally or financially sustain social distancing was 14 days or longer, given the 14-day recommended time to self-isolate if you test positive for COVID-19. These variables take the value 0 if their stated maximum time was 13 days or less. Table 2 shows that 84% of participants stated that they can afford to continue their current level of social distancing for 14 days or more, while 87% stated that they can emotionally tolerate another 14 days or more of their current level of social distancing.

The variable *Lifestyle impact – healthy* is an index that measures the extent to which social distancing has changed participants’ behavior in a healthier direction. Participants were assigned a value 1 for each of the following: if they stated that social distancing had (a) increased consumption of vegetables, (b) decreased in-between-meals snacking (excluding fruits and vegetables), (c) increased time spent in green spaces, (d) increased time spent doing strenuous or (e) moderate exercising and (f) reduced stress. This variable could take a value between 0 and 6, where higher values represent healthier changes. The variable *Lifestyle impact – unhealthy* is an index that measures the extent to which social distancing has changed behavior in an unhealthy direction. Participants were assigned a value 1 for each of the following: if they stated that social distancing had (a) decreased consumption of vegetables, (b) increased in-between-meals snacking (excluding fruits and vegetables), (c) decreased time spent in green spaces, (d) decreased time spent doing strenuous or (e) moderate exercising and (f) increased stress. This variable could also take a value between 0 and 6, where higher values represent a higher number of unhealthy changes. The summary statistics in Table 2 suggest that social distancing has led to more unhealthy behavior than it has healthy behavior, as implied by the lower mean value of *Lifestyle impact – healthy*. We note, however, that these variables are crude measures of the lifestyle impact from social distancing, where each change is given equal weight, although some changes might have a more important health effect than others.^1^

The variable *Business impact* takes a value 1 if a participant is a business owner whose business has experienced negative impacts due to COVID-19, such as their operation losing income, going out of business or being at risk of going out of business. The variable *Employer impact* takes a value 1 if a participant is an employee and his/her employer has experienced a negative impact due to COVID-19, such as their employer losing income or being at risk for going out of business, if they had experienced pay cuts, reduced working hours or were on unpaid leave as a result of the virus.^2^ Table 2 shows that of participants who are business owners, or where until a month ago (*n* = 89), 64% had experienced a negative impact on their business from COVID-19. Of participants who are employees, or were until a month ago (*n* = 477), 57% had experienced a negative impact on their job security, payment or employer revenues.

The variable *Social distant compliant – 6 feet* measures how many people a participant has been close to in the last 3 days. Participants could state ‘none’, 1 person, 2–3 people, 4–5 people, 6–9 people, 10–15 people, 16–25 people, 26–50 people or ‘more than 50 people’. We assigned participants the midpoint of the range they picked. For those in the highest range (50 and more), we assumed the same size interval as the second to highest interval (i.e., we assumed an endpoint of the last interval equal to 75 people). Table 2 shows that the average number of people that participants had been close to during the last 3 days, besides their household members, was 5.76. Although not reported in the Table 2, the median was 2.5.

The variable *Social distant compliant – groups* measures how many times during the last 3 days a participant has been in a room with 10 or more people. Participants could state a value anywhere between zero and ‘5 or more times’. The median of this variable is 0. Table 2 shows that participants on average had been in a room with 10 or more people around 0.5 times during the last 3 days.

The variable *Self health risk* measures the sum of 10 underlying health conditions that would put the participant at higher risk for developing severe health consequences if becoming infected with COVID-19. These health conditions include chronic respiratory conditions, heart disease, neurological conditions, diabetes and obesity (CDC, 2020). The variable *Child health risk* measures the same sum of underlying health conditions for a child in the household. The variable *Worry about own health* is based on the stated extent to which participants worry about their own health due to COVID-19, where the value 0 indicates ‘not at all’ and the value 3 indicates ‘a lot’.

The dummy variable *Insurance* takes a value 1 if a participant states that he/she has private health insurance or is covered by Medicare or Medicaid and 0 if the participant stated not having any coverage. Table 2 shows that 69% of participants have insurance or Medicare or Medicaid coverage. The dummy variables *Republican*, *Democrat* and *Other political party* take a value 1 if a participant identifies as Republican, Democrat or neither, and 0 otherwise. About 34% of participants identify as Republican, 43% as Democrat and 23% as other.

The variable *Extrovert* is based on the extraversion scale developed by Francis *et al.* (1992) and includes participants’ answers to questions such as “Are you a talkative person?” and “Can you easily get some life into a rather dull party?” In addition to the extraversion scale, we also asked participants to indicate their level of agreement with statements about their general social lifestyle, such as “My social life is very important to me,” and “In my spare time, my favorite thing to do is to spend time with friends.” If the participant answered yes to three or more of these questions, they were assigned a 1 for the *Extrovert* dummy variable. The answers to these statements were, however, highly correlated with the extraversion scale, so they were excluded from our analysis because they provided little or no additional information.

## Results

When we pool participants from both treatments who had not been tested prior to participating in our study (*n* = 897), we find that 69% of participants would be willing to take a costless COVID-19 test.^3^ We find no difference in shares of participants willing to test across treatments (Pearson χ^2^ (1.619); p = 0.203), suggesting that the location of self-isolation (at home or in a facility away from home), in the event the test comes back positive, is not an important determinant of people's willingness to test for COVID-19. Not only is the treatment effect small, it is also of the unexpected sign – the share of people willing to test if self-isolation would happen at home is smaller (67%) than the share of people willing to test if self-isolation might happen at a facility away from home (71%). If anything, people might be slightly more inclined to test if a positive result could lead to isolation away from home (potentially due to this also signaling the greater severity of the COVID-19 situation), but the size of the effect is too small for us to detect with our sample size. We have ruled out that this absence of identifiable average effect masks any potentially ‘rational’ heterogeneity in the population (i.e., we have explored whether there exists a treatment effect for subgroups of the population, such as those with children, higher-quality homes (as measured by income) or health anxiety (as measured by underlying health conditions)). One interpretation of the absence of treatment effect is that people assign little weight to the personal cost associated with the location of self-isolating when they decide on whether to take a COVID-19 test.

We pool participants from both treatments and examine the determinants of willingness to test. We estimate a probit model. Table 3 shows the resulting average marginal effects.

**Table 3.** Determinants of willingness to test for COVID-19.

Variable	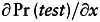
Female	–0.059
	(0.030)
Emotional tolerance	0.063
	(0.051)
Financial tolerance	–0.037
	(0.049)
Lifestyle impact – healthy	0.030**
	(0.013)
Lifestyle impact – unhealthy	0.018**
	(0.009)
Business impact	0.074
	(0.066)
Employer impact	–0.049
	(0.034)
High-risk age	–0.126***
	(0.041)
Rural area	–0.018
	(0.031)
Social distance compliant – 6 feet	–0.003**
	(0.002)
Social distance compliant – groups	–0.015
	(0.017)
Self health risk	0.020
	(0.013)
Child health risk	–0.027
	(0.037)
Insurance	0.082**
	(0.035)
Extrovert	0.087**
	(0.029)
Republican	–0.127***
	(0.034)
Other political party	–0.059
	(0.039)
Worry about own health	0.063***
	(0.015)
Observations	890

Standard errors in parentheses.

*p < 0.1, **p < 0.05, ***p < 0.01.

Concerns about own health are captured by the variable *Worry about own health*. The results in Table 3 imply that the more a person worries about their health due to COVID-19, the more likely they are to take a test. The inclusion of this variable in our model also renders the coefficient for the variable that measures underlying health conditions (i.e., *Self health risk*) small and statistically insignificant (if *Worry about own health* is excluded from the regression, *Self health risk* has the expected positive, and statistically significant, effect on willingness to test). We do not find that people with children who have underlying health conditions are more likely to take the test, perhaps due to the expectation that people of young age are less affected. This result remains robust if we recode the variable *Child health risk* into a dummy variable that takes the value 1 if any child in the household has one or more underlying health conditions.

Variables that affect a person's financial situation do not seem to matter to the willingness to take a COVID-19 test. In particular, we do not find an effect from *Business impact* or *Employer impact*. We examine the robustness of these findings to alternative measures of business and employer impact. First, we instead include the multitude of variables underlying *Business impact* and *Employer impact* in the regression model (see Footnote 2), but we do not find an effect from any of those variables that is close to statistically significant at even the 10% level. Furthermore, we do not find an effect on willingness to test from *Financial tolerance*. Second, we recode the variables such that they range from little impact to severe impact (ranging from if a business owner or employee has experienced no adverse effects from COVID-19, to one or multiple effects). Again, we find no statistically significant effects from these variables on the willingness to test. Taken together, these results suggest that people do not consider their own private costs from a positive test when deciding on taking a COVID-19 test. Similarly, we find no effect on the willingness to test from *Emotional tolerance*, implying that the private emotional cost from social isolation in the event of a positive test might not affect the decision to take a COVID-19 test.

We find that healthy younger people are more likely to take a test than healthy older people, as implied by the negative parameter estimate for *High-risk age*. This age effect is consistent with findings in other studies that observe older people avoid health-related information more than younger people (Thunström *et al.*, 2016; Gigerenzer & Garcia-Retamero, 2017). While this result could imply that older people are more likely to be willfully ignorant, it is also in line with the idea that those with more social interactions (younger people) are more likely to get tested. Studies find that people below 60 years old have more social contacts, and therefore are more likely to transmit infectious diseases (Mossong *et al.*, 2008). Furthermore, we find that those who have met more people during the last 3 days are more willing to take the test, as suggested by the negative parameter estimate for *Social distance compliant – 6 feet*. Furthermore, the potential ‘super-spreaders’ – the extroverts – are more likely (by 8%) to take a test compared to the introverts. Taken together, this suggests that social benefits weight heavily in people's decisions to test; those most at risk to spread COVID-19 are the most willing to get tested.

We find that Republicans are 13% less likely than Democrats to get tested. We speculate that this might be due to different information sources and because the risks of COVID-19 might be portrayed differently in liberal and conservative popular and social media. We examined the robustness of this result by including a conservatism scale (Everett, 2013) in the probit regression, and the result remains the same: people who are more conservative are less likely to want to take a COVID-19 test. The conservatism scale is, however, not included in the final model, given its high correlation with the political dummy variables.

Finally, we find that people with health insurance, or coverage from Medicare or Medicaid, are around 8% more likely to take the test. This result might suggest that people who lack health care coverage use willful ignorance as a means to reduce anxiety about how to deal with a diagnosis. This would be in line with previous studies that suggest willful ignorance of health diagnoses may be motivated by the drive to reduce anxiety about the future (e.g., Oster *et al.*, 2013).

Our results are robust to the inclusion of other explanatory variables, such as race, education, income and profession with high exposure to infected people (health care worker, store clerk, etc.). But these variables lack explanatory power or are highly correlated with other explanatory variables included in Table 3.

The tests conducted prior to participating in our study were neither randomly offered to people (so far, testing for COVID-19 in the USA has been primarily of individuals who showed symptoms), nor costless (52% of those who had tested prior to participating in our study stated the tests were financially costly and 60% said testing was time consuming). The value of data on observed testing is limited when it comes to helping us understand whether people might purposefully ignore such tests. While acknowledging that, we still compare our identified determinants of testing in Table 3 to the determinants of having taken a test before participating in our study (see Online Supplementary Material). While the levels of statistical significance vary, all coefficients are of the same sign as those in Table 3, except for four variables. Having taken a test before participating in our study seems to be positively affected by having spent more time in groups with 10 or more people (i.e., *Social distance compliant – groups*), as well as by a child having underlying health conditions (i.e., *Child health risk*). Employer impact has a (weakly) statistically significant positive effect on testing prior to participating in our study, while it is not statistically significant in Table 3. *Insurance* is not a statistically significant determinant of having been tested prior to participating in our study, while it does have an effect in Table 3.

## Discussion

Widespread testing is one of the most important actions that US governments at any level can undertake to help slow down the spread of COVID-19. Given budget and testing supply constraints, it is likely that random, but voluntary, testing will be the most effective policy. We design a survey to examine the risks from self-selection into taking a COVID-19 test.

Overall, we observe that around 70% of people would agree to a costless COVID-19 test. We find that people who are more worried about their own health due to COVID-19 are more likely to test, as are young healthy people, relative to older healthy people. Ability to afford self-isolation for 14 days does not seem to affect the decision to test. Furthermore, people who worry more about their health, and people with health insurance or health coverage through Medicare or Medicaid, are more likely to take the test, as are people identifying as Democrats compared to Republicans.

Contrary to our expectation, we also find that potential ‘super-spreaders’ are *more* likely than other individuals to agree to a costless COVID-19. It could be that extroverts are more willing than expected to take a COVID-19 test because their private cost of doing so is unusually low due to the broadly implemented social distancing at the time of data collection for this study. If extroverts are already relatively isolated (i.e., due to a stay-at-home order and mandated closures by the state governor of public spaces, such as gyms, restaurants and bars), the personal cost of testing might be low. Furthermore, extroverts might be more likely to get infected if they socialize more, which could be a ‘selfish’ motivation to get tested. However, we control for the current level of compliance with social distancing, which should address both of these private motivations for increased probability of testing, and we find that people who comply more are *less* motivated to take the test. We also control for their worry about own health due to COVID-19. Even so, the positive effect on willingness to test from being an extrovert persists. We therefore conclude that the positive effect of being an extrovert on willingness to test for COVID-19 is likely due to social health benefits weighing more heavily in their decision than their private costs from potential self-isolation for 14 days, should the test come back positive. The importance of the prosocial motive in determining COVID-19 testing is consistent with the results of the study by Jordan *et al.* (2020), who find that prosocial messages are more effective than self-interested messages in promoting behavior that prevent the spread of COVID-19 (e.g., hand washing, hand shaking, hugging).

Our results suggest that the risks of adverse selection (in terms of failing to target the people most likely to spread the virus) in testing for COVID-19 might be fairly low. This underscores the value of widespread testing, even if it cannot be truly random, and the importance of making such testing available nationwide in the USA as soon as possible.

An important shortcoming of our analysis is that it builds on hypothetical survey data. It is well documented that survey answers may be affected by a ‘hypothetical bias’, meaning that people answer one way in a survey and behave in a different way when faced with real, incentivized decisions. This risk pertains to our study as well, and the hypothetical bias might be particularly pronounced if the choice to test for COVID-19 is regarded as prosocial. Several studies suggest that a hypothetical bias is particularly likely when measuring prosocial behavior – people often exaggerate the extent to which they engage in such behavior (e.g., Murphy *et al.*, 2005; Vossler *et al.*, 2012; Jacquemet *et al.*, 2013). Furthermore, it is possible that personal costs to the testing decision are less salient in a hypothetical context. Once testing is more widespread in the USA, it will be important to examine who actually chooses to get tested, and the extent to which they deviate from the general population. That said, hypothetical and incentivized behavior generally correlate, such that an analysis like ours can provide important insights into the potential pitfalls of voluntary testing, prior to the actual testing. This is useful information to have on hand when designing an efficient and cost-effective testing strategy.

## Footnotes

1 Participants could also state that social distancing had not changed their behavior in any direction, which prevents *Lifestyle impact – healthy* and *Lifestyle impact – unhealthy* from being perfectly collinear.

2 More specifically, these variables are generated from participants’ answers to how their business or employer had been impacted by COVID-19. A total of 53% of business owners stated that their business was at risk of going out of business; 23% of business owners stated that their business had gone out of business; and 70% of business owners stated that their income had declined. Of employees, 29% said that their employer was at risk of going out of business; 16% of workers said that their employer had gone out of business; 52% of workers said that their employer had seen their revenues decline; 20% of workers said that they had lost their job; 27% of workers said that they had been placed on unpaid leave; and 23% of workers said that they had had their pay reduced.

3 Participants who stated that they would prefer not to take the test (278/897) were asked for their reasons not to want to take the test. They were given the following alternatives and were asked to mark all that apply: “I would not change my behavior if I learned I had the virus” (11%); “I do not want to self-isolate for 14 days” (4%); “My job prevents me from self-isolating for 14 days” (6%); “I think I have already had the virus” (2%); “It would cause me emotional discomfort if I knew I had the virus” (7%); “It doesn't matter to me if I get tested or not” (30%); other (53%). The numbers in parentheses show the shares of participants who agreed with the statement. As shown, a large share of participants stated that the test would not matter, and that they would not change their behavior anyway. A potential reason for the prominence of these reasons could be that they are already highly complying with social distancing. (Table 3 shows that the more people comply with social distancing, the less likely they are to want to take a COVID-19 test.) However, a large share also states ‘other’, suggesting that the alternatives presented to our participants did not cover the full range of reasons as to why people may refrain from testing. We encourage future research to further explore these reasons.

## Supplementary material

For supplementary material accompanying this paper visit https://doi.org/10.1017/bpp.2020.15. click here to view supplementary material

## References

[ref1] Center for Disease Control and Prevention (CDC) (2020), What You Can Do if You Are at Higher Risk of Severe Illness from COVID-19. https://www.cdc.gov/coronavirus/2019-ncov/downloads/COVID19-What-You-Can-Do-High-Risk.pdf, Retrieved April 4, 2020.

[ref2] Chandler, J. and G. Paolacci (2017), ‘Lie for a dime: When most prescreening responses are honest but most study participants are impostors’, Social Psychological and Personality Science, 8(5): 500–508.

[ref3] Conrads, J. and B. Irlenbusch (2013), ‘Strategic ignorance in ultimatum bargaining’, Journal of Economic Behavior & Organization, 92: 104–115.

[ref4] COVID Tracking Project (2020), Most recent data. https://covidtracking.com/data Retrieved April 30 2020.

[ref5] Cutler, S. J. and L. G. Hodgson (2003), ‘To test or not to test: Interest in genetic testing for Alzheimer's disease among middle-aged adults’, American Journal of Alzheimer's Disease & Other Dementias®, 18(1): 9–20. 10.1177/153331750301800106 PMC10833794 12613129

[ref6] Dana, J., R.A. Weber and J. Xi Kuang, (2007), ‘Exploiting moral wiggle room: experiments demonstrating an illusory preference for fairness’, Economic Theory, 33(1): 67–80.

[ref7] Everett, J. A. (2013), ‘The 12 item social and economic conservatism scale (SECS)’, PloS one, 8(12). 10.1371/journal.pone.0082131 PMC3859575 24349200

[ref8] Francis, L. J., L. B. Brown and R. Philipchalk (1992), ‘The development of an abbreviated form of the Revised Eysenck Personality Questionnaire (EPQR-A): Its use among students in England, Canada, the USA and Australia’, Personality and individual differences, 13(4): 443–449.

[ref9] Ganguly, A. R. and J. Tasoff (2017), ‘Fantasy and dread: The demand for information and the consumption utility of the future’, Management Science, 63 (12): 4037–4060.

[ref10] Gigerenzer, G. and R. Garcia-Retamero (2017), ‘Cassandra's regret: the psychology of not wanting to know’, Psychological Review, 124(2): 179. 28221086 10.1037/rev0000055

[ref11] Grossman, Z. (2014), ‘Strategic Ignorance and the Robustness of Social Preferences’, Management Science, 60(11): 2659–2665.

[ref12] Grossman, Z. and J. van der Weele (2017), ‘Self-image and willful ignorance in social decisions’, Journal of the European Economic Association, 15(1): 173–217.

[ref13] Harvard Medical School (2020), Coronavirus Resource Center. https://www.health.harvard.edu/diseases-and-conditions/coronavirus-resource-center Retrieved March 28, 2020.

[ref14] Hightow, L. B., W. C. Miller, P. A. Leone, D. Wohl, M. Smurzynski and A. H. Kaplan (2003), ‘Failure to return for HIV posttest counseling in an STD clinic population’, AIDS Education and Prevention, 15(3): 282–290. 12866839 10.1521/aeap.15.4.282.23826

[ref15] Jacquemet, N., R. V. Joule, S. Luchini and J. F. Shogren (2013), ‘Preference elicitation under oath’, Journal of Environmental Economics and Management, 65(1): 110–132.

[ref16] Jordan, J.J., E. Yoeli and D.G. Rand (2020), Don't get it or don't spread it? Comparing self-interested versus prosocially framed COVID-19 prevention messaging, *Working paper*, https://psyarxiv.com/yuq7x 10.1038/s41598-021-97617-5 PMC8511002 34642341

[ref17] Mossong, J., N. Hens, M. Jit, P. Beutels, K. Auranen, R. Mikolajczyk, M. Massari, S. Salmaso, G. Scalia Tomba, J. Wallinga, J. Heijne, M. Sadkowska-Todys, M. Rosinska, W.J. Edmunds (2008), ‘Social contacts and mixing patterns relevant to the spread of infectious diseases’, PLoS Medicine, 5(3). doi:10.1371/journal.pmed.0050074 PMC2270306 18366252

[ref18] Murphy, J. J., P. G. Allen, T. H. Stevens and D. Weatherhead (2005), ‘A meta-analysis of hypothetical bias in stated preference valuation’, Environmental and Resource Economics, 30(3): 313–325.

[ref19] Nordström, J., L. Thunström, K. van't Veld, J.F. Shogren and M. Ehmke (2020), ‘Strategic ignorance of health risk: Its causes and policy consequences’, Behavioural Public Policy, 1–32. Doi:10.1017/bpp.2019.52

[ref20] Onwezen, M. C. and C. N. van der Weele (2016), ‘When indifference is ambivalence: strategic ignorance about meat consumption’, Food Quality and Preference, 52, 96–105.

[ref21] Oster, E., I. Shoulson and E. R. Dorsey (2013), ‘Optimal expectations and limited medical testing: Evidence from Huntington disease’, American Economic Review, 103(2): 804–30. 10.1257/aer.106.6.1562 29547253

[ref22] Sharot, T. and C. R. Sunstein (2020), ‘How people decide what they want to know’, Nature Human Behaviour, 4, 14–19. Doi: 10.1038/s41562-019-0793-1 31932690

[ref23] Sharpe Wessling, K., J. Huber and O. Netzer (2017), ‘MTurk character misrepresentation: Assessment and solutions’, Journal of Consumer Research, 44(1): 211–230.

[ref24] Stigler, G. J. (1961), ‘The economics of information’, Journal of Political Economy, 69(3): 213–225.

[ref25] Stock, J. (2020), Random Testing Is Urgently Needed. http://www.igmchicago.org/wp-content/uploads/2020/03/Random-Testing-is-Urgently-Needed_IGM.pdf

[ref26] Sunstein, C. R. (2019), ‘Ruining popcorn? The welfare effects of information’, Journal of Risk and Uncertainty, 58(2–3): 121–142.

[ref27] Thompson, H. S., H. B. Valdimarsdottir, C. Duteau-Buck, J. Guevarra, D. H. Bovbjerg, C. Richmond-Avellaneda and K. Offit (2002), ‘Psychosocial predictors of BRCA counseling and testing decisions among urban African-American women’, Cancer Epidemiology and Prevention Biomarkers, 11(12): 1579–1585. 12496047

[ref28] Thunström, L., J. Nordström, J. F. Shogren, M. Ehmke and K. van't Veld (2016), ‘Strategic self-ignorance’, Journal of Risk and Uncertainty, 52(2): 117–136.

[ref29] Thunström, L. (2019), ‘Welfare effects of nudges: The emotional tax of calorie menu labeling’, Judgment and Decision making, 14(1): 11.

[ref30] Vossler, C. A., M. Doyon and D. Rondeau (2012), ‘Truth in consequentiality: theory and field evidence on discrete choice experiments’, American Economic Journal: Microeconomics, 4(4): 145–71.

[ref31] Woolley, K. and J. L. Risen (2018), ‘Closing your eyes to follow your heart: Avoiding information to protect a strong intuitive preference’, Journal of Personality and Social Psychology, 114(2): 230. 29251947 10.1037/pspa0000100

[ref32] World Health Organization (WHO) (2020). Report of the WHO-China Joint Mission on Coronavirus Disease 2019 (COVID-19). https://www.who.int/docs/default-source/coronaviruse/who-china-joint-mission-on-covid-19-final-report.pdf

