# Supplementary material for: Testing for COVID-19: willful ignorance or selfless behavior?
Source: Behav Public Policy. 2020 May 8:1–18. doi: 10.1017/bpp.2020.15 (PMC7256418; doi:10.1017/bpp.2020.15)
Supplement: Supplementary file 1 [file S2398063X20000159sup001.docx]

**Online** **Supplementary Material**

**Testing for COVID-19:**

**Willful ignorance or selfless behavior?**

Linda Thunström^1*^, Madison Ashworth^1^, Jason F. Shogren^1^,

Stephen Newbold^1^ and David Finnoff^1^

^1^Department of Economics, University of Wyoming, 82071 Laramie WY, USA.

*Corresponding author. Department of Economics, University of Wyoming, 82071 Laramie WY, USA. Email: lthunstr@uwyo.edu.

**Descriptive statistics of participants who had tested for COVID-19 prior to survey participation**

Of our total sample of 1000 participants, 103 participants stated they had already been tested for COVID-19 before participating in the survey. Of these, we lack information about one participant’s employment status. That participant is dropped from the probit regression (Table A2) and also from the descriptive statistics in Table A1.

**Table A1**. Summary statistics of participants who had tested for COVID-19 prior to participating in study

| **Variable** | Obs | Mean | Std. Dev | Min | Max |
| --- | --- | --- | --- | --- | --- |
|  |  |  |  |  |  |
| Female | 102 | .4216 | .4962 | 0 | 1 |
|  |  |  |  |  |  |
| Emotional tolerance | 102 | .7549 | .4323 | 0 | 1 |
|  |  |  |  |  |  |
| Financial tolerance | 102 | .6863 | .4663 | 0 | 1 |
|  |  |  |  |  |  |
| Lifestyle impact - Healthy | 102 | 2.471 | 1.621 | 0 | 5 |
|  |  |  |  |  |  |
| Lifestyle impact – Unhealthy | 102 | 2.441 | 1.428 | 0 | 6 |
|  |  |  |  |  |  |
| Business Owner | 102 | .3627 | .4832 | 0 | 1 |
|  |  |  |  |  |  |
| Employee | 102 | .5196 | .5021 | 0 | 1 |
|  |  |  |  |  |  |
| Unemployed | 102 | .1176 | .3238 | 0 | 1 |
|  |  |  |  |  |  |
| Business impact | 37 | .2941 | .4579 | 0 | 1 |
|  |  |  |  |  |  |
| Employer impact | 53 | .4216 | .4962 | 0 | 1 |
|  |  |  |  |  |  |
| High risk age | 102 | .0294 | .1698 | 0 | 1 |
|  |  |  |  |  |  |
| Rural area | 102 | .1373 | .3458 | 0 | 1 |
|  |  |  |  |  |  |
| Social distance compliant – 6ft | 102 | 5.236 | 7.819 | 0 | 38 |
|  |  |  |  |  |  |
| Social distance compliant – groups | 102 | 1.275 | 1.260 | 0 | 5 |
|  |  |  |  |  |  |
| Self health risk | 102 | 1.412 | 2.266 | 0 | 10 |
|  |  |  |  |  |  |
| Child health risk |  | .8235 | 2.099 | 0 | 10 |
|  |  |  |  |  |  |
| Insurance | 102 | .4706 | .5016 | 0 | 1 |
|  |  |  |  |  |  |
| Republican | 102 | .3431 | .4771 | 0 | 1 |
|  |  |  |  |  |  |
| Democrat | 102 | .5392 | .5009 | 0 | 1 |
|  |  |  |  |  |  |
| Other Political Party | 102 | .1176 | .3238 | 0 | 1 |
|  |  |  |  |  |  |
| Extrovert | 102 | .6078 | .4906 | 0 | 1 |
|  |  |  |  |  |  |
|  |  |  |  |  |  |
| Age | 102 | 34.32 | 11.09 | 18 | 68 |
|  |  |  |  |  |  |
| Worry own health | 102 | 1.853 | 1.054 | 0 | 3 |

**Determinants of testing for COVID-19 prior to survey participation**

**Table A2**. Determinants of testing for COVID-19 prior to study participation – average marginal effects from a probit regression.

| **Variable** | $\partial\Pr\left( test \right)/\partial x$ | |  |
| --- | --- | --- | --- |
|  | |  | |
| Female | | -0.010 | |
|  | | (0.017) | |
| Emotional tolerance | | 0.001 | |
|  | | (0.024) | |
| Financial tolerance | | -0.051** | |
|  | | (0.022) | |
| Lifestyle impact – Healthy | | 0.033*** | |
|  | | (0.006) | |
| Lifestyle impact – Unhealthy | | 0.009 | |
|  | | (0.006) | |
| Business impact | | 0.073** | |
|  | | (0.026) | |
| Employer impact | | 0.027* | |
|  | | (0.019) | |
| High risk age | | -0.072* | |
|  | | (0.040) | |
| Rural area | | -0.072*** | |
|  | | (0.021) | |
| Social distance compliant – 6ft | | -0.002** | |
|  | | (0.001) | |
| Social distance compliant – groups | | 0.024*** | |
|  | | (0.008) | |
| Self health risk | | 0.006 | |
|  | | (0.005) | |
| Child health risk | | 0.038*** | |
|  | | (0.010) | |
| Insurance | | -0.021 | |
|  | | (0.018) | |
| Extrovert | | 0.032* | |
|  | | (0.017) | |
| Republican | | -0.012 | |
|  | | (0.019) | |
| Other Political Party | | -0.026 | |
|  | | (0.023) | |
| Worry about own health | | .0100 | |
|  | | (0.008) | |
| Observations | 992 | |  |

Standard errors in parentheses. *** p<0.01, ** p<0.05, * p<0.1.

**Survey questions pertaining to the study**

Start of Block: Intro question and consent

Welcome to our study,
  
 You have been invited to participate in a survey. You must be at least 18 years of age to participate.

If you agree to participate in this survey you will be asked about your health related attitudes and decisions. Your participation may last up to 10 min.

There are minimal risks involved with participating in this study that do not go beyond those of everyday life. Participation in this study is voluntary. You may refuse to participate or withdraw at any time.

No personal identifiers will be collected in this study. Until completion of the study, the data collected in this study will be stored in a password protected folder accessible only by the research team. After the study has been completed, the data and results from this study will be shared with the wider scientific community, in line with the guidelines from the top scientific journals. Following the preferences of these journals, we will do so by storing the data in an open access scientific database. The shared data will be in a form that contains no identification of the participants in this study.    

Please undertake the study in private, keeping distractions to a minimum (phone, computer, TV, etc.).

If you have questions about the study, please contact any of the below participants in the research group.

Researcher contact information:      
Madison Ashworth, Economics, University of Wyoming, phone: XXXX, mashwor1@uwyo.edu

Linda Thunstrom, Economics, University of Wyoming, phone: XXXX, lthunstr@uwyo.edu

Please indicate below if you consent to participating in this study.

- Yes, I consent.
- No, I do not consent.

End of Block: Intro question and consent

Start of Block: Screening questions

Which gender do you identify with?

- Male
- Female

What is your age?

- 17 or less
- 18-34
- 35-54
- 55+

What is your race?

- Non-Hispanic White
- African American
- Hispanic
- Other

What is your highest level of education?

- High School/GED or Less
- Some College
- College Graduate
- Graduate Degree

| 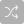 |
| --- |

What is your individual annual, pre-tax, income (including bonuses and commissions) in U.S. dollars?

- $24,999 or less
- $25,000 - $49,999
- $50,000 - $99,999
- $100,000 or more

| 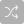 |
| --- |

What is your household annual, pre-tax, income (including bonuses and commissions) in U.S. dollars?

- $24,999 or less
- $25,000 - $49,999
- $50,000 - $99,999
- $100,000- $149,999
- $150,000 - $199,99
- $200,001 or more

In what region of the United States do you reside?

- Midwest
- Northeast
- South
- West

End of Block: Screening questions

Start of Block: Info about coronavirus

The world is currently experiencing an outbreak of a new coronavirus (COVID-19). In this study, we want to learn about your attitudes towards this new coronavirus.

Please answer this survey as truthfully as you can.

There are no right or wrong answers, but your honest views and opinions are important and will help advance our knowledge about the potential public response to the coronavirus and similar viruses that may emerge in the future.

- I assure that I read the above text
- I did not read the above text

| Page Break |  |
| --- | --- |

According to the World Health Organization, there are 209,839 confirmed cases of COVID-19 and 8,778 deaths globally. In the United States alone there were 15,219 cases and 201 reported deaths on March 20 (source: Centers for Disease Control and Prevention, CDC).

The CDC estimates the risk of death for people who get infected. Those who have preexisting medical conditions like high blood pressure, heart disease, or lung disease are at higher risk of developing severe illness from COVID-19 (WHO). Further, risks vary with age. The below table shows the risk of death from COVID-19 if getting infected, across ages.

For instance, of those age 85 and above who contract the virus, 10-17% will likely die, while currently no one under the age of 19 who contracted the virus has died. 

- I assure that I read the above text
- I did not read the above text

End of Block: Info about coronavirus

Start of Block: Tested for the coronavirus

Have you been tested for the coronavirus (COVID-19)?

- Yes, I have
- No, I have not

End of Block: Tested for the coronavirus

***If answered “yes” to having been tested***:

Start of Block: Reasons for having been tested

What was the primary reason you were tested for the coronavirus?

- I showed symptoms of the coronavirus
- A family member showed symptoms of the coronavirus
- A friend showed symptoms of the coronavirus
- I showed no symptoms, but wanted to know if I was infected
- A family member was tested positive for the coronavirus
- A friend was tested positive for the coronavirus
- Other

Are you currently infected with the coronavirus?

- Yes
- No
- I do not want to share this information

|  |
| --- |

How many days ago were you tested?

________________________________________________________________

To what extent do you agree with the statements below in regards to the coronavirus test?

|  | Yes | No |
| --- | --- | --- |
| It was financially costly to get tested. |  |  |
| It was very time consuming to get tested. |  |  |

End of Block: Reasons for having been tested

***If answered “no” to having been tested***:

Start of Block: Wanted test or not

Have you tried to get tested for the coronavirus (COVID-19)?

- No
- Yes

What was the primary reason you tried to get tested for the coronavirus?

- I showed symptoms of the coronavirus
- A family member showed symptoms of the coronavirus
- A friend showed symptoms of the coronavirus
- I showed no symptoms, but wanted to know if I was infected
- A family member was tested positive for the coronavirus
- A friend was tested positive for the coronavirus
- Other

|  |
| --- |

How many days ago did you try to get tested?

________________________________________________________________

End of Block: Wanted test or not

Start of Block: TREATMENT 1 testing with self quarantine

**Currently, U.S. authorities are working to test more people for the coronavirus.**   **Legislators are urging people who test positive, i.e, are found to be infected by the virus, to self-isolate at home for 14 days.**

**Self-isolation means:**

Stay at home.
Do not go to work. 
Consider working remotely if that is an option until the 14-day period has ended.
Avoid all non-essential travel around your community.
Avoid crowds, public events, meetings, social activities, or other group activities.
You may go shopping for food or take a walk outside.
Do not share utensils, toothbrushes, water bottles, pillows, and avoid shaking hands, kissing, hugging, or other intimate activities. 
Avoid close contact. (Close contact is defined as closer than a 6-foot distance between you and others.) 

 (Source: <https://yalehealth.yale.edu/self-isolation-information>)

**If you were given the opportunity to take a coronavirus test for free within the next 3 days, would you take the test?**

- Yes, I would take the test
- No, I would not take the test

Please let us know why you prefer not to take the coronavirus test. Mark all that apply.

- I would not change my behavior if I learned I had the virus.
- I do not want to self-isolate for 14 days.
- My job prevents me from self-isolating for 14 days.
- I think I have already had the virus.
- It would cause me emotional discomfort if I knew I had the virus.
- It doesn't matter to me if I get tested or not.
- Other

End of Block: TREATMENT 1 testing with self quarantine

Start of Block: TREATMENT 2 testing with removing oneself

**Currently, U.S. authorities are working to test more people for the coronavirus.  Legislators are urging people who test positive, i.e, are found to be infected by the virus to self-isolate for 14 days. Some states have started building self-quarantine sites -- sites where people who have the virus would be isolated for 14 days.**

**If people stay at those sites, it is easier to ensure they comply with the guidelines to self-isolate.**

**Self-isolation means**:
Stay at home.
Do not go to work. 
Consider working remotely if that is an option until the 14-day period has ended.
Avoid all non-essential travel around your community.
Avoid crowds, public events, meetings, social activities, or other group activities.
You may go shopping for food or take a walk outside.
Do not share utensils, toothbrushes, water bottles, pillows, and avoid shaking hands, kissing, hugging, or other intimate activities. 
Avoid close contact. (Close contact is defined as closer than a 6-foot distance between you and others.) 

(Source: <https://yalehealth.yale.edu/self-isolation-information>)

**If you were given the opportunity to take a coronavirus test for free within the next 3 days, would you take the test?**

- Yes, I would take the test
- No, I would not take the test

Please let us know why you prefer not to take the coronavirus test. Mark all that apply.

- I would not change my behavior if I learned I had the virus.
- I do not want to self-isolate for 14 days.
- My job prevents me from self-isolating for 14 days.
- I think I have already had the virus.
- It would cause me emotional discomfort if I knew I had the virus.
- It doesn't matter to me if I get tested or not.
- Other

End of Block: TREATMENT 2 testing with removing oneself

Start of Block: Current social distancing

Think back on the last 3 days. Please provide the number of people (outside of your household) whom you have been physically closer to than 6 feet.

- None
- 1 person
- 2-3 people
- 4-5 people
- 6-9 people
- 10-15 people
- 16-25 people
- 26-50 people
- More than 50

Think back on the last 3 days. Please provide the number of times you were in a room with a group larger than 10 people (please exclude visits to the supermarket or pharmacy, to get essential food or medicine).

- I have not been in a room with more than 10 people during the last 3 days
- Once
- Twice
- Three times
- Four times
- Five or more times

To slow down the rate of coronavirus infections in the U.S., state, local and federal governments have undertaken a range of measures to reduce social distance -- so called "social distancing" policies. 
  
 These policies mean travel restrictions, that schools, universities, and daycare centers all over the country have temporarily ceased operations, cultural events have been canceled, tourist attractions including Broadway and Disney World have closed, and national sports leagues have suspended or canceled their seasons.

 These policies also entail recommendations that urge citizens to avoid gatherings of 10 or more people, stay home with their family, and avoid close contact with others (keep a distance to other people of minimum 6 feet), at least for a few weeks, maybe for longer. Please choose the alternative below that best represents your adaptation of these recommendations.

- I fully comply with these recommendations, and do all I can to minimize contact with any people outside of my household.
- I comply with these recommendations -- I stay home as much as I can and make sure not to mix with groups of more than 10 people.
- I somewhat comply with these recommendations -- I stay home for the most part and mostly avoid mixing with groups of more than 10 people.
- I involuntarily comply with the recommendations, because the opportunities to interact with other people are limited.
- I coincidentally comply with the recommendations, because I do not like to interact with other people.
- I do not comply with the recommendations, and seek social interaction with other people.
- I do not comply with the recommendations, because my job prevents me from doing so.

For how much longer do you think that you can afford to sustain your current level of social distancing (i.e., how long until you would need to start looking for a source of income that may alter your current level of social distancing)?

- Not even a day
- Another few days
- Another week
- Another 2 weeks
- Another 3 weeks
- Another 4 weeks
- Another 5 weeks
- Another 6 weeks
- Another couple of months
- Another half a year or more

For how long do you think that you can emotionally sustain your current level of social distancing?

- Not even a day
- Another few days
- Another week
- Another 2 weeks
- Another 3 weeks
- Another 4 weeks
- Another 5 weeks
- Another 6 weeks
- Another couple of months
- Another half a year or more

| Page Break |  |
| --- | --- |

How has your current level of social distancing affected your intake of fruit and vegetables?

- Social distancing has increased my intake of fruit and vegetables
- Social distancing has decreased my intake of fruit and vegetables
- Social distancing has not affected my intake of fruit and vegetables

How has your current level of social distancing affected your intake of snacks (EXCLUDING fruits and vegetables) in between meals (chips, crackers, candy, etc)?

- Social distancing has increased my intake of snacks
- Social distancing has decreased my intake of snacks
- Social distancing has not affected my intake of snacks

How has your current level of social distancing affected your time spent in green spaces outdoors (e.g., city parks, backyard, wilderness)?

- Social distancing has increased my time spent in green spaces
- Social distancing has decreased my time spent in green spaces
- Social distancing has not affected my time spent in green spaces

How has your current level of social distancing affected your time spent doing strenuous exercising (e.g., aerobics, swimming laps, calisthenics, running, jogging, basketball, cycling on hills, racquetball)?

- Social distancing has increased my time spent doing strenuous exercising
- Social distancing has decreased my time spent doing strenuous exercising
- Social distancing has not affected my time spent doing strenuous exercising

How has your current level of social distancing affected your time spent doing moderate exercising (e.g., brisk walking, golf, volleyball, cycling on level streets, recreational tennis, and softball)?

- Social distancing has increased my time spent doing moderate exercising
- Social distancing has decreased my time spent doing moderate exercising
- Social distancing has not affected my time spent doing moderate exercising

How has your current level of social distancing affected your stress level?

- Social distancing has increased my stress level
- Social distancing has decreased my stress level
- Social distancing has not affected my stress level

End of Block: Plans to maintain social distancing

Start of Block: Agreement with policy

To slow down the rate of coronavirus infections in the U.S., state, local and federal governments have undertaken a range of "social distancing" measures and policies. 
 
These policies entail travel restrictions to Canada, China, Iran, and 26 European countries to reduce external exposure to the virus. These policies also mean that schools, universities, and daycare centers all over the country have temporarily ceased operations, cultural events have been canceled, tourist attractions have closed, and national sports leagues have suspended or canceled their seasons. 

 
People are recommended to stay home with their family, avoid gatherings of 10 people or more and avoid close contact with others (keep a distance to other people of minimum 6 feet).
 
Do you agree or disagree with these social distancing policies? 

- I agree with the recommendations, we need to adopt social distancing
- I disagree with the recommendations, we should not be adopting social distancing
- I don't know

You answered that you think social distancing should be adopted. What is the primary reason you agree with social distancing?

- Social distancing is essential to slow down the virus and prevent our medical system from being overwhelmed
- I trust the opinion of the public health experts who believe we need to adapt social distancing
- I worry about the health of myself and/or close family members if being exposed to the coronavirus
- I trust the opinion of the legislators who believe we need to adapt social distancing
- Other

You answered that you do not think social distancing should be adopted. What is the primary reason you disagree with social distancing?

- Social distancing will not to slow down the virus enough to prevent our medical system from being overwhelmed
- Social distancing is too costly given its impact on unemployment, local businesses, etc.
- I worry about the economic consequences of social distancing on my household
- Other

| Page Break |  |
| --- | --- |

To what extent do you think social distancing is worth it?

|  | True | False |
| --- | --- | --- |
| I believe the health benefits from social distancing outweigh the economic consequences for me personally |  |  |
| I believe the health benefits from social distancing outweigh the economic consequences for my family |  |  |
| I believe the health benefits from social distancing outweigh the economic consequences for society as a whole |  |  |
| If I only think about myself, I would prefer to skip the social distancing and face the higher health risk from doing so |  |  |

Start of Block: Main provider

How many adults (18 years and older) live in your household (including yourself)?

- One
- Two
- Three
- Four
- Five or more

Who is the main income provider in your household?

- Me
- The contributions of me and my partner are equally important to our household income
- My partner
- Neither of the above

End of Block: Main provider

Start of Block: Employment of main provider who is you

Please mark the alternative that applies to you.

- I am a business owner (or was until a month ago)
- I am an employee (or was until a month ago)
- I have been unemployed for more than a month

|  | True | False | I do not know |
| --- | --- | --- | --- |
| My business is unaffected by the economic downturn from the coronavirus |  |  |  |
| My business is at risk of going out of business due to the economic downturn from the coronavirus |  |  |  |
| My business has gone out of business due to the economic downturn from the coronavirus |  |  |  |
| The revenue flow of my business has declined substantially due to the economic downturn from the coronavirus |  |  |  |

|  | True | False | I do not know |
| --- | --- | --- | --- |
| My employer is unaffected by the economic downturn from the coronavirus |  |  |  |
| My employer is at risk of going out of business due to the economic downturn from the coronavirus |  |  |  |
| My employer has gone out of business due to the economic downturn from the coronavirus |  |  |  |
| The revenue flow of my employer has declined substantially due to the economic downturn from the coronavirus |  |  |  |
| I recently lost my job due to the economic downturn from the coronavirus |  |  |  |
| I am on unpaid leave due to the economic downturn from the coronavirus |  |  |  |
| I am on reduced pay leave due to the economic downturn from the coronavirus |  |  |  |

To what extent is the following true for your job?

|  | Disagree | Somewhat disagree | Neither agree/disagree | Somewhat agree | Agree |
| --- | --- | --- | --- | --- | --- |
| If I would be on leave (due to sickness) for two weeks, I would likely lose my job |  |  |  |  |  |
| My employer would be ok with me being away for two weeks due to sickness |  |  |  |  |  |
| I could not afford to be absent from work for two weeks |  |  |  |  |  |
| I could take a pay cut for two weeks due to sickness, and still be financially stable |  |  |  |  |  |
| I would be financially stable for a decent period of time, even if I lost my job |  |  |  |  |  |

End of Block: Employment of main provider who is you

Start of Block: Employment of main provider

Please mark the alternative that applies to the main income provider in your household.

- The main income provider in our household is a business owner (or was until a month ago)
- The main income provider in our household is an employee (or was until a month ago)
- The main income provider in our household has been unemployed for more than a month

|  | True | False | I do not know |
| --- | --- | --- | --- |
| The main provider's business is unaffected by the economic downturn from the coronavirus |  |  |  |
| The main provider's business is at risk of going out of business due to the economic downturn from the coronavirus |  |  |  |
| The main provider's business has gone out of business due to the economic downturn from the coronavirus |  |  |  |
| The revenue flow of the main provider's business has declined substantially due to the economic downturn from the coronavirus |  |  |  |

|  | True | False | I do not know |
| --- | --- | --- | --- |
| The main provider's employer is unaffected by the economic downturn from the coronavirus |  |  |  |
| The main provider's employer is at risk of going out of business due to the economic downturn from the coronavirus |  |  |  |
| The main provider's employer has gone out of business due to the economic downturn from the coronavirus |  |  |  |
| The revenue flow of the main provider's employer has declined substantially due to the economic downturn from the coronavirus |  |  |  |
| The main provider recently lost his/her job due to the economic downturn from the coronavirus |  |  |  |
| The main provider is on unpaid leave due to the economic downturn from the coronavirus |  |  |  |
| The main provider is on reduced pay leave due to the economic downturn from the coronavirus |  |  |  |

To what extent is the following true for the job of the main income provider in your household (who might be you)?

|  | Disagree | Somewhat disagree | Neither agree/disagree | Somewhat agree | Agree |
| --- | --- | --- | --- | --- | --- |
| If he/she would be on leave (due to sickness) for two weeks, he/she would likely lose the job |  |  |  |  |  |
| His/her employer would be ok with him/her being away for two weeks due to sickness |  |  |  |  |  |
| He/she could not afford to be absent from work for two weeks |  |  |  |  |  |
| He/she could take a pay cut for two weeks due to sickness, and still be financially stable |  |  |  |  |  |
| He/she would be financially stable for a decent period of time, even if he/she lost his/her job |  |  |  |  |  |

Please mark all of the below that apply to the main income provider in your household.

|  | Yes | No |
| --- | --- | --- |
| Has a chronic respiratory disease (e.g., asthma) |  |  |
| Has cardiovascular disease |  |  |
| Has had adverse reactions to a flu vaccine. |  |  |
| Has a neurological condition |  |  |
| Pregnant |  |  |
| Obese |  |  |
| Has diabetes |  |  |
| Has HIV/AIDS |  |  |
| Has cancer |  |  |
| Has heart disease |  |  |
| Has high bloodpressure |  |  |
| Has health care insurance |  |  |
| Is on Medicare or Medicaid |  |  |
| Is 65 years or older |  |  |

End of Block: Employment of main provider

Start of Block: Risk factors for contracting the virus

Please mark all of the below that apply to you:

- Live in a densely populated area
- Live in a rural area
- Live in a nursing home
- Live in a long-term care facility
- Live by myself
- Live with my children (at least part of the time)
- Travel extensively abroad
- I work in a health care facility
- I am a teacher
- I am a firefighter
- I work in a store that sells essential goods, such as groceries, pharmaceuticals or gas

End of Block: Risk factors for contracting the virus

Start of Block: Risk factors in household

How many children (age 18 and under) live in your household?

- No children live in my household
- One child lives in my household
- Two or more children live in my household

|  | Yes | No |
| --- | --- | --- |
| Have a chronic respiratory disease (e.g., asthma) |  |  |
| Have cardiovascular disease |  |  |
| Have had adverse reactions to a flu vaccine. |  |  |
| Have a neurological condition |  |  |
| Pregnant |  |  |
| Obese |  |  |
| Have diabetes |  |  |
| Have HIV/AIDS |  |  |
| Have cancer |  |  |
| Have heart disease |  |  |
| Have high blood pressure |  |  |
| Have health care insurance |  |  |
| Am on Medicare or Medicaid |  |  |

|  |
| --- |

How old is your child (in years)?

________________________________________________________________

Please mark all of the below that apply to you or your child:

|  | Myself | My child | Doesn't apply to anyone |
| --- | --- | --- | --- |
| Have a chronic respiratory disease (e.g., asthma) |  |  |  |
| Have cardiovascular disease |  |  |  |
| Have had adverse reactions to a flu vaccine. |  |  |  |
| Have a neurological condition |  |  |  |
| Pregnant |  |  |  |
| Obese |  |  |  |
| Have diabetes |  |  |  |
| Have HIV/AIDS |  |  |  |
| Have cancer |  |  |  |
| Have heart disease |  |  |  |
| Have high blood pressure |  |  |  |
| Have health care insurance |  |  |  |
| Are on Medicare or Medicaid |  |  |  |
| Have tested positive for the coronavirus (COVID-19) |  |  |  |

How many children live in your household?

________________________________________________________________

|  |
| --- |

How old is your youngest child (in years)?

________________________________________________________________

|  |
| --- |

How old is your oldest child (in years)?

________________________________________________________________

Please mark all of the below that apply to you or any of your children:

|  | Myself | My youngest child | My oldest child | Does not apply to anyone |
| --- | --- | --- | --- | --- |
| Have a chronic respiratory disease (e.g., asthma) |  |  |  |  |
| Have cardiovascular disease |  |  |  |  |
| Have had adverse reactions to a flu vaccine. |  |  |  |  |
| Have a neurological condition |  |  |  |  |
| Pregnant |  |  |  |  |
| Obese |  |  |  |  |
| Have diabetes |  |  |  |  |
| Have HIV/AIDS |  |  |  |  |
| Have cancer |  |  |  |  |
| Have heart disease |  |  |  |  |
| Have high blood pressure |  |  |  |  |
| Have health care insurance |  |  |  |  |
| Have Medicare or Medicaid |  |  |  |  |
| Have tested positive for the coronavirus (COVID-19) |  |  |  |  |

End of Block: Risk factors in household

Start of Block: Perceived risks

To what extent do you worry about the following, due to the coronavirus?

|  | Not at all | Somewhat | Quite a bit | A lot |
| --- | --- | --- | --- | --- |
| My own health |  |  |  |  |
| The health of my family members |  |  |  |  |
| My own financial situation |  |  |  |  |
| The financial situation of my family members |  |  |  |  |

End of Block: Perceived risks

Start of Block: Extraversion, Francis et al, 1992, abbreviated form of Eysenck personality quest

|  | Yes | No |
| --- | --- | --- |
| Are you a talkative person? |  |  |
| Are you rather lively? |  |  |
| Can you easily get some life into a rather dull party? |  |  |
| Do you tend to keep in the background on social occasions? |  |  |
| Are you mostly quiet when you are with other people? |  |  |
| Do other people think of you as being very lively? |  |  |

To what extent do you agree with the below?

|  | Disagree | Somewhat disagree | Neither agree/disagree | Somewhat agree | Agree |
| --- | --- | --- | --- | --- | --- |
| My social life is very important to me |  |  |  |  |  |
| In my spare time, my favorite thing to do is to spend time with friends |  |  |  |  |  |
| In my spare time, my favorite thing to do is to spend time with family |  |  |  |  |  |
| I almost always prefer to go out and meet people, over staying at home |  |  |  |  |  |
| I really value time to myself |  |  |  |  |  |
| I can go for days without seeing anybody, and still feel great |  |  |  |  |  |

End of Block: Extraversion, Francis et al, 1992, abbreviated form of Eysenck personality quest

Start of Block: Demographic questions

|  |
| --- |

What is your age?

________________________________________________________________

What is your living situation?

- I live in an apartment
- I live in a single-family home
- I live in a townhouse

Do you have easy access (short drive or walk) to green spaces? (e.g., city parks, backyard, wilderness)

- Yes
- No
- I do not know

| Page Break |  |
| --- | --- |

What is your religious preference?

- Jewish
- Muslim
- Mormon
- Protestant
- Catholic
- Buddhist
- Hindu
- None -- I identify with atheism
- None -- I identify with agnosticism
- Other

Do you believe in God?

- Yes
- No
- I am not sure

How often do you attend religious services?

- Never
- Once or twice a year
- Less than once a month
- Once a month
- 2-3 times a month
- Once a week
- Twice a week or more

What is your marital status?

- Single, never married
- Married or domestic Partnership
- Widowed
- Divorced

Do you think of yourself as closer to the Democratic or the Republican party?

- I'm closer to the Democratic party
- I'm closer to the Republican party
- I don't think of myself as closer to either of them

Please indicate the extent to which you feel positive or negative towards each issue. Scores of 0 indicate greater negativity, and scores of 100 indicate greater positivity. Scores of 50 indicate that you feel neutral about the issue.

|  | 0 | 10 | 20 | 30 | 40 | 50 | 60 | 70 | 80 | 90 | 100 |
| --- | --- | --- | --- | --- | --- | --- | --- | --- | --- | --- | --- |

| Abortion | 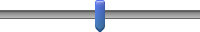 |
| --- | --- |
| Limited government | 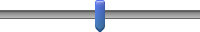 |
| Military and national security | 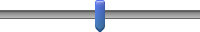 |
| Religion | 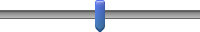 |
| Welfare benefits | 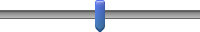 |
| Gun ownership | 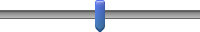 |
| Traditional marriage | 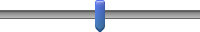 |
| Traditional values | 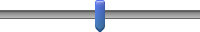 |
| Fiscal responsibility | 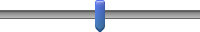 |
| Business | 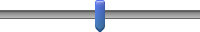 |
| The family unit | 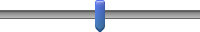 |
| Patriotism | 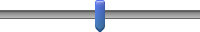 |

What is your favorite color? Regardless of what your favorite color is, **please state orange AND green below**. This question is to ensure the survey is not being taken by a bot.

- Yellow
- Blue
- Red
- Orange
- Purple
- Green
- Blue
- Pink
- Brown

End of Block: Demographic questions

**End survey.**
